# Human papillomavirus DNA and p16 expression in Japanese patients with oropharyngeal squamous cell carcinoma

**DOI:** 10.1002/cam4.151

**Published:** 2013-10-27

**Authors:** Hisato Kawakami, Isamu Okamoto, Kyoichi Terao, Kazuko Sakai, Minoru Suzuki, Shinya Ueda, Kaoru Tanaka, Kiyoko Kuwata, Yume Morita, Koji Ono, Kazuto Nishio, Yasumasa Nishimura, Katsumi Doi, Kazuhiko Nakagawa

**Affiliations:** 1 Department of Medical Oncology, Kinki University Faculty of Medicine377-2 Ohno-higashi, Osaka-Sayama, Osaka, 589-8511, Japan; 2 Center for Clinical and Translational Research, Kyushu University Hospital3-1-1 Maidashi, Higashiku, Fukuoka, 812-8582, Japan; 3 Department of Otolaryngology, Kinki University Faculty of Medicine377-2 Ohno-higashi, Osaka-Sayama, Osaka, 589-8511, Japan; 4 Department of Genome Biology, Kinki University Faculty of Medicine377-2 Ohno-higashi, Osaka-Sayama, Osaka, 589-8511, Japan; 5 Radiation Oncology Research Laboratory, Research Reactor Institute, Kyoto UniversitySennan-gun, Osaka, 590-0494, Japan; 6 Department of Radiation Oncology, Kinki University Faculty of Medicine377-2 Ohno-higashi, Osaka-Sayama, Osaka, 589-8511, Japan

**Keywords:** DNA methylation, human papillomavirus, oropharynx, p16, squamous cell carcinoma

## Abstract

Human papillomavirus (HPV) is a major etiologic factor for oropharyngeal squamous cell carcinoma (OPSCC). However, little is known about HPV-related OPSCC in Japan. During the study, formalin-fixed, paraffin-embedded OPSCC specimens from Japanese patients were analyzed for HPV DNA by the polymerase chain reaction (PCR) and for the surrogate marker p16 by immuno-histochemistry. For HPV DNA-positive, p16-negative specimens, the methylation status of the p16 gene promoter was examined by methylation-specific PCR. Overall survival was calculated in relation to HPV DNA and p16 status and was subjected to multivariate analysis. OPSCC cell lines were examined for sensitivity to radiation or cisplatin in vitro. The study results showed that tumor specimens from 40 (38%) of the 104 study patients contained HPV DNA, with such positivity being associated with tumors of the tonsils, lymph node metastasis, and nonsmoking. Overall survival was better for OPSCC patients with HPV DNA than for those without it (hazard ratio, 0.214; 95% confidence interval, 0.074–0.614; *P* = 0.002). Multivariate analysis revealed HPV DNA to be an independent prognostic factor for overall survival (*P* = 0.015). Expression of p16 was associated with HPV DNA positivity. However, 20% of HPV DNA-positive tumors were negative for p16, with most of these tumors manifesting DNA methylation at the p16 gene promoter. Radiation or cisplatin sensitivity did not differ between OPSCC cell lines positive or negative for HPV DNA. Thus, positivity for HPV DNA identifies a distinct clinical subset of OPSCC with a more favorable outcome in Japanese.

## Introduction

Head and neck cancer is the sixth most common cancer worldwide, with an estimated annual incidence of approximately 600,000 cases [1]. Although the incidence of such cancer overall has fallen in recent years, consistent with the decrease in tobacco use, that of oropharyngeal squamous cell carcinoma (OPSCC) has increased in both the United States and Europe. In 2009, the International Agency for Research on Cancer recognized human papillomavirus (HPV) type 16 as a causal agent of OPSCC [2]. Individuals with HPV-positive OPSCC show significantly better overall survival and disease-free survival, associated with a 20–80% reduction in the risk of death, compared with those with HPV-negative OPSCC [3, 4]. Knowledge of HPV status in patients with OPSCC is thus expected to play an increasing role in the management of this disease. Epidemiological evidence from several countries indicates that the proportion of OPSCC cases caused by HPV varies widely, however. Although the proportion of OPSCC cases attributable to HPV ranges from 40 to 80% in the United States and is around 90% in Sweden [3, 5], little is known about HPV-related OPSCC in Asian populations.

The aim of this study was to evaluate the prevalence, clinical features, and outcome of OPSCC positive for HPV DNA in the Japanese population. We also assessed the concordance between the presence of HPV DNA in tumor specimens and expression of the host cyclin-dependent kinase inhibitor p16 as detected by immunohistochemistry (IHC), given that p16 is commonly examined as a surrogate marker for HPV positivity in OPSCC [3, 6], and we further investigated possible mechanisms underlying any discordance. Moreover, to evaluate the biological impact of HPV infection, we examined the sensitivity of OPSCC cell lines positive or negative for HPV DNA to radiation and to cisplatin.

## Material and Methods

### Patients and tissue

**Figure 1:**
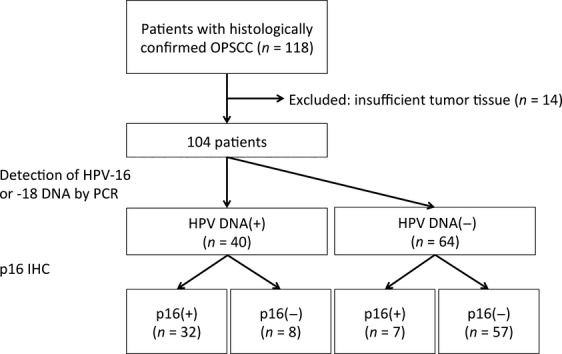
Summary of the protocol for classification of enrolled OPSCC patients according to HPV DNA and p16 status. OPSCC, oropharyngeal squamous cell carcinoma; HPV, human papillomavirus.

With approval of the appropriate institutional review board, we analyzed formalin-fixed, paraffin-embedded (FFPE) tissue from 118 consecutive patients with newly diagnosed and histologically confirmed OPSCC who were treated at Kinki University Hospital from November 2000 through December 2011. Tumor specimens for all cases were obtained during surgery or diagnostic biopsy, and one representative paraffin block was selected for each case. Several 6-μm paraffin sections were used for analysis of HPV DNA, and one 3-μm section was used for p16 IHC. Patients without sufficient tumor tissue available for both analysis of HPV DNA and p16 staining were excluded, leaving 104 patients in the study (Fig. 1).

Clinicopathologic characteristics and outcome data for patients were obtained from the medical records. Treatment modality was selected for each patient individually on the basis of the official published guidelines. Most individuals underwent radiation therapy or radio-chemotherapy according to a standard fractionated regimen, receiving 60–70 Gy with or without concomitant platinum-based chemotherapy. Adjuvant radiotherapy (54–64 Gy) was administered with standard fractionation.

### Analysis of HPV DNA

The FFPE specimens were depleted of paraffin and then subjected to macrodissection in order to select a region of cancer tissue. Genomic DNA was extracted from the cancer tissue with the use of a QIAamp DNA Micro Kit (Qiagen, Hilden, Germany), and the DNA concentration of each extract was determined with a NanoDrop 2000 spectrophotometer (Thermo Scientific, Waltham, MA). DNA for HPV types 16, 18, 31, 33, and 35 was detected with the use of a TaqMan real-time polymerase chain reaction (PCR)-based method (Applied Biosystems, Foster City, CA) that was designed to amplify the E6 region or E7 region (or both) of the viral genome. The primer and probe sequences for amplification have been described previously [7–9]. Samples of genomic DNA that had sufficient amplifiable β-globin DNA (>1 human genome/μL) were considered to be evaluable, and HPV type was determined for β-globin gene-positive and HPV DNA-positive specimens. We defined active HPV DNA involvement as PCR detection at the level of at least one copy per 10 cell genomes [7]. PCR analysis was performed in duplicate.

### IHC for detection of p16 expression

**Figure 2:**
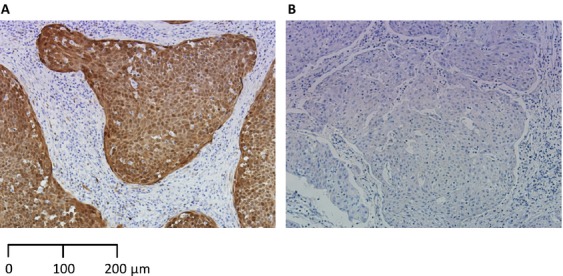
Representative IHC staining of p16 in OPSCC tumor specimens. Tumors were classified as either positive (A) or negative (B) for p16 expression. Scale bar, 200 μm. IHC, immunohistochemistry; OPSCC, oropharyngeal squamous cell carcinoma.

Immunohistochemistry for p16 was performed with the use of a CINtec Histology Kit (MTM Laboratories AG, Heidelberg, Germany) based on the monoclonal antibody E6H4. A tonsil squamous cell carcinoma with a high level of p16 expression was used as a positive control, and the primary antibody was omitted as a negative control. Expression of p16 was scored positive if strong and diffuse nuclear and cytoplasmic staining was present in >70% of the tumor cells [10], and p16 scoring was performed without knowledge of HPV status. Representative p16 IHC images are shown in Figure 2.

### Methylation-specific-PCR analysis

For assessment of DNA methylation at the p16 gene promoter, genomic DNA samples were subjected to sodium bisulfite modification with the use of a MethylEasy Xceed Rapid DNA Bisulfite Modification Kit (Human Genetic Signatures, Randwick, NSW, Australia). The modified DNA was then used as a template for methylation-specific (MS)-PCR with primers specific for methylated or unmethylated sequences [11]. The sizes of the MS-PCR products were previously described [12]. Real-time MS-PCR analysis was performed in a 25-μL reaction mixture with the use of an EpiScope MSP Kit (Clontech, Mountain View, CA). EpiScope Methylated HCT116 gDNA and EpiScope Unmethylated HCT116 DKO gDNA (Clontech) were used as positive and negative controls, respectively.

### Cell culture

The human OPSCC cell lines UPCI-SCC-003, UPCI-SCC-036, UPCI-SCC-089, UPCI-SCC-090, UPCI-SCC-152, and UPCI-SCC-154 were kindly provided by S. Gollin (University of Pittsburgh School of Public Health, Pittsburgh, PA). The cells were cultured under an atmosphere of 5% CO_2_ at 37°C in Dulbecco's modified Eagle's medium (Sigma-Aldrich, St Louis, MO) supplemented with 10% heat-inactivated fetal bovine serum (Hyclone, Logan, UT), 0.1 mmol/L nonessential amino acids (Gibco-Invitrogen, Carlsbad, CA), 2 mmol/L l-glutamine, and 1 mmol/L sodium pyruvate (Sigma-Aldrich).

### Clonogenic survival assay

Exponentially growing cells in 25-cm^2^ flasks were harvested by exposure to trypsin and counted. They were diluted serially to appropriate densities, plated in triplicate in 25-cm^2^ flasks containing 10 mL of complete medium, and exposed at room temperature to various doses of radiation with a ^60^Co irradiator at a rate of ∼0.82 Gy/min. The cells were cultured for 14–21 days, fixed with methanol:acetic acid (10:1, v/v), and stained with crystal violet. Colonies containing >50 cells were counted. The surviving fraction was calculated as: (mean number of colonies)/(number of plated cells × plating efficiency). Plating efficiency was defined as the mean number of colonies divided by the number of plated cells for corresponding nonirradiated cells.

### Cell growth inhibition assay

Cells were transferred to 96-well flat-bottomed plates and cultured for 24 h before exposure to various concentrations of cisplatin for 72 h. Cell Counting Kit-8 solution (Dojindo, Kumamoto, Japan) was then added to each well, and the cells were incubated for 3 h at 37°C before measurement of absorbance at 490 nm with a Multiskan Spectrum instrument (Thermo Labsystems, Boston, MA). Absorbance values were expressed as a percentage of that for nontreated cells, and the median inhibitory concentration (IC_50_) of cisplatin for inhibition of cell growth was determined.

### Statistical analysis

Patient characteristics were compared between individuals positive or negative for HPV DNA with Student's two-tailed *t*-test or the chi-square test. Survival curves were constructed by the Kaplan–Meier method and were compared with the log-rank test. The impact of various factors on survival was evaluated by multivariate analysis according to the Cox regression model. Concordance between HPV DNA and p16 assay results was assessed with the kappa statistic (κ) and Spearman correlation. Statistical analysis was performed with the use of IBM SPSS statistics software version 20 (SPSS Inc., IBM, Chicago, IL). A *P*-value of <0.05 was considered statistically significant.

## Results

### Patient characteristics

The characteristics of the 104 studied patients are listed in Table 1. The median age of the patients was 64 years, with a range from 35 to 80 years, and most of them were male patients (78%) and had stage IV disease (74%).

**Table 1:** Characteristics of the 104 study patients according to HPV DNA and p16 status

		HPV DNA(+) (*n*=40, 38%), *n* (%)		HPV DNA(−) (*n*=64, 62%), *n* (%)		
				
	All patients (*n*=104), *n* (%)	p16(+) (*n*=32)	p16(−) (*n*=8)	p16(+) (*n*=7)	p16(−) (*n*=57)	HPV DNA(+) vs. HPV DNA(−) (*P*-value)
Sex						
Male	81 (78)	23 (72)	6 (75)	5 (71)	47 (82)	0.329
Female	23 (22)	9 (28)	2 (25)	2 (29)	10 (18)	
Age (years)						
Median	64	60	66	71	65	0.276
Range	35–80	36–75	35–71	59–77	38–80	
T classification						
1–2	70 (67)	23 (72)	6 (75)	6 (86)	35 (61)	0.372
3–4	34 (33)	9 (28)	2 (25)	1 (14)	22 (39)	
N classification						
0	29 (28)	3 (9)	3 (38)	1 (14)	22 (39)	0.021
1–3	75 (72)	29 (91)	5 (63)	6 (86)	35 (61)	
Stage						
I–III	27 (26)	6 (19)	3 (38)	1 (14)	17 (30)	0.524
IV	77 (74)	26 (81)	5 (63)	6 (86)	40 (70)	
Tobacco usage						
Never smoker	39 (38)	21 (66)	1 (13)	5 (71)	14 (25)	0.010^1^
<40 pack-years	27 (26)	9 (28)	2 (25)	0 (0)	16 (28)	
>40 pack-years	38 (37)	2 (6)	5 (63)	2 (29)	27 (47)	
Tumor location						
Tonsil	60 (58)	26 (81)	5 (63)	5 (71)	24 (42)	0.002^2^
Posterior wall	6 (6)	0 (0)	0 (0)	0 (0)	6 (11)	
Lateral wall	4 (4)	2 (6)	0 (0)	1 (14)	1 (2)	
Base of tongue	13 (13)	4 (13)	0 (0)	1 (14)	8 (14)	
Anterior palatine arch	13 (13)	0 (0)	2 (25)	0 (0)	11 (19)	
Unknown	8 (8)	0 (0)	1 (13)	0 (0)	7 (12)	
Initial therapy^3^						
RT(+)	85 (82)	27 (84)	8 (100)	7 (100)	43 (75)	0.426
RT(−)	19 (18)	5 (16)	0 (0)	0 (0)	14 (25)	

*P*-values were calculated with Student's two-tailed *t*-test for age and the chi-square test for other variables. HPV, human papillomavirus.

1 Comparison of patients who never smoked versus patients with a smoking history.

2 Comparison between tonsil and other sites.

3 RT(+), treatment with radiation, including radiation therapy alone (*n*=3), chemoradiotherapy alone (*n*=13), or surgery followed by radiation therapy (*n*=46) or by chemoradiotherapy (*n*=23); RT(−), treatment without radiation, including surgery alone (*n*=11), surgery followed by chemotherapy (*n*=1), chemotherapy alone (*n*=4), and best supportive care (*n*=3).

### Presence of HPV DNA and p16 expression in OPSCC

Of the 104 tumor specimens, 40 (38%) were positive for HPV-16 or HPV-18 DNA by PCR analysis (Fig. 1). These 40 tumors included 37 positive for HPV-16 alone, two positive for HPV-18 alone, and one positive for both HPV-16 and HPV-18. HPV DNA was detected more frequently in the tonsils (*P* = 0.002) than in other regions (Table 1). Patients positive for HPV DNA presented significantly more often with lymph node metastasis (85 vs. 64%, *P* = 0.021) and included a higher proportion of never-smokers (55 vs. 30%, *P* = 0.010) compared with those negative for HPV. There was no significant association between HPV DNA status and gender, age, T classification, or disease stage.

Expression of p16 was detected by IHC in a total of 39 tumors (Fig. 2). Of the 40 cases positive for HPV DNA, 32 (80%) were positive for p16, whereas 57 (89%) of the 64 cases negative for HPV DNA were also negative for p16 (Fig. 1). There was thus good agreement between HPV DNA positivity and p16 positivity (κ = 0.65; 95% confidence interval [CI], from 0.50 to 0.80; *r* = 0.631; *P* < 0.001).

### DNA methylation at the p16 gene promoter in OPSCC

**Figure 3:**
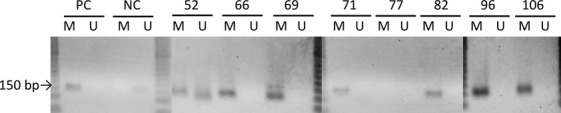
MS-PCR analysis of the p16 gene promoter in eight OPSCC tumors positive for HPV DNA but negative for p16 by IHC. The position of a 150-bp amplification product corresponding to the methylated promoter is indicated. PC, positive control; NC, negative control; M, methylated; U, unmethylated; MS-PCR, methylation-specific polymerase chain reaction; OPSCC, oropharyngeal squamous cell carcinoma; HPV, human papillomavirus; IHC, immunohistochemistry.

To identify the underlying mechanism of p16 gene silencing in tumors positive for HPV DNA but negative for p16 expression, we examined the DNA methylation status of the p16 gene promoter region with the use of MS-PCR analysis. Among the eight such cases, DNA methylation at the p16 gene promoter was detected in six (cases 66, 69, 71, 82, 96, and 106) (Fig. 3).

### Survival analysis

**Figure 4:**
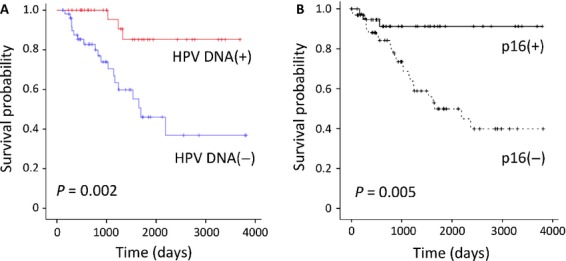
Kaplan–Meier curves for overall survival of OPSCC patients according to HPV DNA (A) or p16 (B) status. *P*-values were calculated by the log-rank test. OPSCC, oropharyngeal squamous cell carcinoma; HPV, human papillomavirus.

Oropharyngeal squamous cell carcinoma patients positive for HPV DNA showed a significantly better overall survival compared with those negative for HPV DNA [hazard ratio (HR), 0.214; 95% CI, from 0.074 to 0.614; *P* = 0.002] (Fig. 4A). For OPSCC of stages I to III, HPV-positive patients tended to have a better overall survival compared with their HPV-negative counterparts, but the difference was not statistically significant (*P* = 0.129), possibly because of the small sample size (*n* = 27) (Fig. S1A). On the other hand, for OPSCC of stage IV (*n* = 77), patients with HPV DNA showed a significantly better overall survival than did those without it (*P* = 0.002) (Fig. S1B). Stratification based on p16 expression also revealed a significantly better outcome for OPSCC patients positive for p16 than for those negative for this marker (HR, 0.245; 95% CI, from 0.085 to 0.705; *P* = 0.005) (Fig. 4B). To rule out potential confounding effects for the presence of HPV DNA and other factors, we performed multivariate analysis for overall survival (Table 2). The presence of HPV DNA was revealed to be an independent and significant prognostic factor for overall survival (HR, 0.248; 95% CI, from 0.080 to 0.766; *P* = 0.015) after taking into account gender, age, T and N classification, smoking history, tumor location, and radiation therapy.

**Table 2:** Multivariate analysis of overall survival in patients with OPSCC (*n*=104)

	Overall survival		
	
Factor	HR	95% CI	*P*
HPV DNA (positive vs. negative)	0.248	0.080–0.766	0.015
Gender (female vs. male)	0.870	0.231–2.151	0.539
Age (≤63 vs. >64years)	0.833	0.392–1.770	0.634
T classification (1–2 vs. 3–4)	0.718	0.315–1.640	0.432
N classification (0 vs. 1–3)	1.536	0.640–3.680	0.337
Smoking history (nonsmoker vs. smoker)	0.541	0.120–2.445	0.424
Tumor location (tonsil vs. other)	0.597	0.277–1.289	0.189
RT^1^, RT(+) vs. RT(−)	2.233	0.390–13.89	0.355

OPSCC, oropharyngeal squamous cell carcinoma; HPV, human papillomavirus; HR, hazard ratio; CI, confidence interval.

1 RT(+), treatment with radiation, including radiation therapy alone (*n*=3), chemoradiotherapy alone (*n*=13), or surgery followed by radiation therapy (*n*=46) or by chemoradiotherapy (*n*=23); RT(−), treatment without radiation, including surgery alone (*n*=11), surgery followed by chemotherapy (*n*=1), chemotherapy alone (*n*=4), and best supportive care (*n*=3).

### Sensitivity of OPSCC cell lines with or without HPV DNA to radiation and cisplatin

**Figure 5:**
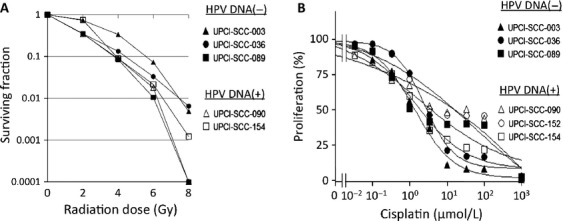
Sensitivity of OPSCC cell lines to radiation or cisplatin according to HPV DNA status. (A) Clonogenic assay for cells exposed to the indicated doses of radiation. This assay was not performed with UPCI-SCC-152 cells. (B) Effect of cisplatin concentration on cell growth. All data are means from three independent experiments. OPSCC, oropharyngeal squamous cell carcinoma; HPV, human papillomavirus.

We next investigated the biological impact of HPV DNA status with OPSCC cell lines positive (UPCI-SCC-090, -152, and -154) or negative (UPCI-SCC-003, -036, and -089) for HPV DNA. A clonogenic survival assay performed after exposure of the cells to various doses of radiation revealed no significant difference in survival between the cell lines positive or negative for HPV DNA (Fig. 5A). We also examined the effect of cisplatin on the growth of the cell lines, again detecting no difference in the IC_50_ value of cisplatin between those positive or negative for HPV DNA (Fig. 5B, Table 3).

**Table 3:** IC_50_ values of cisplatin for inhibition of OPSCC cell growth in vitro

Cell line	HPV DNA	Cisplatin IC_50_ (μmol/L)
UPCI-SCC-003	(−)	1.7
UPCI-SCC-036	(−)	3.0
UPCI-SCC-089	(−)	1.2
UPCI-SCC-090	(+)	5.7
UPCI-SCC-152	(+)	4.6
UPCI-SCC-154	(+)	2.0

IC, inhibitory concentration; OPSCC, oropharyngeal squamous cell carcinoma; HPV, human papillomavirus.

## Discussion

In this study, we applied PCR-based detection of viral DNA and IHC-based detection of p16 to tumor specimens from Japanese patients with OPSCC, given that this combination of approaches is the most reliable means to determine HPV status, with a sensitivity of 97% and specificity of 94% [13]. We found that 38% of the patients were positive for HPV DNA, consistent with recent studies that detected HPV DNA in 30–50% of OPSCC patients in Asian countries [14–16]. In the United States, the incidence of HPV-positive OPSCC increased by 225% from the late 1980s to the early 2000s [17], with 40–80% of OPSCCs now being caused by HPV [3]. This increase is thought to have resulted from the decrease in tobacco use and increased oral HPV exposure due to changes in sexual behavior among recent birth cohorts [3, 4]. As in other Asian countries, the prevalence of smoking in Japan is much higher than that in the United States, especially among men (32 vs. 17%) [18]. The lower proportion of OPSCC cases associated with HPV in Asian countries compared with Western countries might therefore be attributable, at least in part, to the difference in tobacco exposure. Given that the proportion of active smokers has recently been decreasing each year in Japan, the proportion of OPSCCs related to HPV in the Japanese population is likely to increase.

We found that overall survival for Japanese OPSCC patients positive for HPV DNA was significantly better than that for those negative for HPV DNA. The presence of HPV DNA was associated mostly with tumors of the palatine tonsils, lymph node metastasis, and nonsmoking. HPV-positive OPSCC was more frequent in younger individuals than was HPV-negative OPSCC, but the difference was not significant, possibly due to the relatively small sample size. These results are consistent with those for OPSCC in the United States and Europe [3, 4], suggesting similarity in the features of HPV-associated OPSCC between Japan and Western countries.

The reason for the more favorable prognosis of HPV-associated OPSCC remains unclear, although it may be related to a younger age at onset, minimal exposure to established risk factors such as cigarette smoking, or a better response to therapy [3, 19]. Indeed, recent studies have provided evidence that HPV-positive OPSCC shows a better response to chemotherapy [20, 21] or to radiotherapy either alone [22, 23] or in combination with chemotherapy [20, 21, 24, 25]. Although these findings are suggestive of an inherent radio- or chemosensitivity of HPV-positive OPSCC, we did not detect a difference in sensitivity to radiation or cisplatin in vitro between OPSCC cell lines positive or negative for HPV DNA. This apparent discrepancy between the in vitro and clinical data might be due to the limitations of in vitro assays, which do not accurately reflect the tumor microenvironment in vivo. Further study is thus needed to determine the molecular mechanism underlying the favorable outcome of patients with HPV-positive OPSCC, with the prospect that such knowledge might inform the development of therapeutic approaches to improve the poor prognosis of those with HPV-negative OPSCC.

In HPV-positive OPSCC, production of the viral oncoprotein E7 results in inactivation of the retinoblastoma (RB) protein and consequent upregulation of p16 expression [3, 26–28]. IHC positivity for p16 is thus associated with HPV-positive OPSCC, being regarded as a surrogate marker for HPV infection in such tumors [3, 6]. We also found a significant correlation between positivity for HPV DNA and IHC-based detection of p16 in Japanese patients with OPSCC, and the results of survival analysis based on p16 status as a stratification factor were similar to those of such analysis based on HPV DNA status.

Although most HPV-associated OPSCC tumors express p16, we found that 20% of HPV DNA-positive tumors (eight cases) were negative for p16 by IHC. A similar level of discordance was observed in previous studies based on the same approaches for detection of HPV DNA and p16 [7, 13, 29], although the underlying mechanism remains largely unknown. Given that DNA methylation at the p16 gene promoter has been identified as a key mechanism of p16 gene silencing in various types of primary tumor [30], we analyzed the methylation status of the p16 gene promoter in the eight tumors positive for HPV DNA but negative for p16 in this study with the use of MS-PCR analysis. We found a high frequency (6/8, 75%) of DNA methylation at the p16 gene promoter in these cases. As far as we are aware, this is the first demonstration of DNA methylation at the p16 gene promoter in OPSCC tumors positive for HPV DNA but negative for p16 by IHC. A recent meta-analysis showed that heavy cigarette consumption was associated with p16 gene methylation in patients with non-small cell lung cancer [12]. In this study, among the HPV DNA-positive subgroup, patients with tumors negative for p16 expression had a significantly more extensive smoking history than those with tumors positive for p16 (*P* < 0.001, Student's two-tailed *t*-test), suggesting that heavy smoking might be responsible, at least in part, for DNA methylation at the p16 gene promoter and a consequent loss of p16 expression. Consistent with the results of a previous study [7], we also found that the survival of patients with HPV DNA-positive, p16-negative tumors was not as good as that of those with HPV DNA-positive, p16-positive tumors (data not shown). These data thus suggest that IHC-based detection of p16 provides suboptimal prognostic information unless combined with PCR-based detection of HPV DNA.

Seven (11%) of the 64 HPV DNA-negative tumors in this study were positive for p16 by IHC. Given that the HPV DNA analysis was initially restricted to HPV types 16 and 18, we further investigated the possible presence of DNA for other high-risk types of HPV (types 31, 33, and 35), which, together with types 16 and 18, account for most cases of HPV-associated OPSCC [8, 13, 31]. However, none of the seven HPV DNA-negative, p16-positive tumors was found to be positive for these other high-risk types of HPV (data not shown). Similar results have been obtained in previous studies based on detection of HPV by PCR or in situ hybridization [19], with a discordance rate of ∼10–20%. Expression of p16 in such HPV DNA-negative tumors might reflect disturbances of the RB signaling pathway unrelated to HPV infection, as has been found to be the case in malignant lymphoma and small cell lung cancer [32]. The mechanism of p16 expression in the absence of detectable HPV DNA in OPSCC warrants further investigation.

Two prophylactic HPV vaccines against HPV types 6, 11, 16, and 18 (quadrivalent) or HPV types 16 and 18 (bivalent) have shown clinical efficacy for prevention of HPV-related cervical cancer [33] and anal cancer [34]. Both vaccines thus target HPV type 16, which accounts for >90% of HPV-associated OPSCCs [4]. Given the causal relation between HPV infection and OPSCC, clinical evaluation of the potential efficacy of HPV vaccines for reducing the incidence of HPV-associated OPSCC is warranted.

In conclusion, we found that 38% of Japanese patients with OPSCC are positive for HPV DNA, with such positivity being an independent prognostic factor for overall survival. Given that expression of p16 can be affected by genetic or epigenetic changes in addition to HPV infection, our results suggest that IHC-based detection of p16 provides suboptimal prognostic information if not combined with detection of HPV DNA. Further clinical studies are warranted to characterize the mechanism underlying the survival benefit conferred by HPV positivity in patients with OPSCC as well as to identify optimal treatments for this patient population.

## Supporting Information

**Figure S1.** Kaplan–Meier curves for overall survival of patients with OPSCC of stages I to III (A) or of stage IV (B) according to HPV DNA status. *P*-values were calculated by the log-rank test.
