# Supplementary figures and images for: Human papillomavirus DNA and p16 expression in Japanese patients with oropharyngeal squamous cell carcinoma
Source: Cancer Med. 2013 Oct 27;2(6):933–41. doi: 10.1002/cam4.151 (PMC3892398; doi:10.1002/cam4.151)

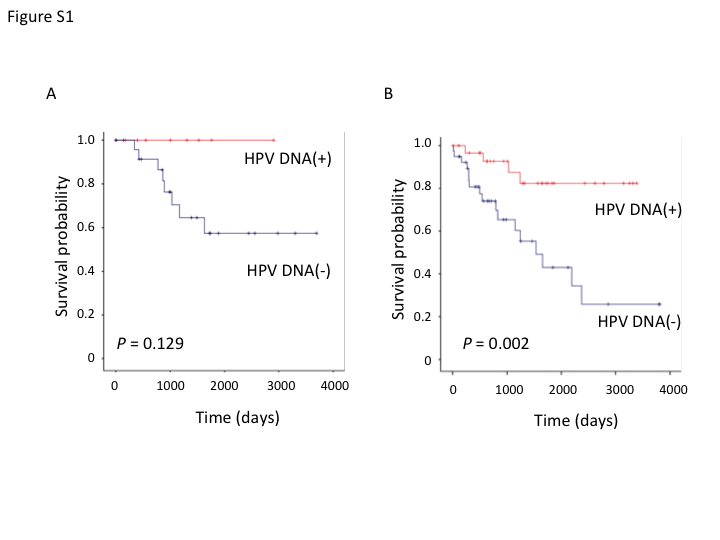

Supplement: Supplementary file 1 [file cam40002-0933-SD1.tiff]
